# Robot-assisted Partial Nephrectomy with the new HugoRAS System: single center matched-pair analysis

**DOI:** 10.1590/S1677-5538.IBJU.2025.0741

**Published:** 2026-07-20

**Authors:** Guilherme Guimarães Gonçalves, Nuno Vinagre, Martinha Magalhães, Avelino Fraga, Miguel Silva-Ramos

**Affiliations:** 1 Unidade Local de Saúde de Santo António Departamento de Urologia Porto Portugal Departamento de Urologia, Unidade Local de Saúde de Santo António, Porto, Portugal

**Keywords:** Kidney Neoplasms, Robotic Surgical Procedures, Minimally Invasive Surgical Procedures

## Abstract

**Purpose:**

Robotic-assisted surgery represents a leading innovation in kidney-sparing procedures across specialized urology centers. As new robotic platforms continue to emerge, comparisons with conventional laparoscopy remain necessary. This study focuses on comparing oncological, perioperative and functional outcomes of robot-assisted partial nephrectomy (RAPN) with the Hugo™ RAS system and laparoscopic partial nephrectomy (LPN) within a tertiary institution.

**Materials and Methods:**

A retrospective single-center study included 165 patients who underwent LPN (n=95) or RAPN (n=70). Perioperative clinical data, tumor characteristics, oncological and functional outcomes were obtained. Propensity score matching (1:1) was implemented to mitigate selection bias. Trifecta and pentafecta were assessed as composite surgical success endpoints. Multivariate logistic regression analyses were performed to identify the predictive factors for each.

**Results:**

Following propensity score matching, 70 patients were included in each group. Operative time, WIT and estimated blood loss did not differ significantly. RAPN was associated with a shorter length of stay (3 days vs 5 days, p<0.001) without increased complication rate. Renal functional outcomes, including ≥90% eGFR preservation and CKD upstaging, were similar. Trifecta (LPN 84.3% vs. RAPN 85.7%) and pentafecta (LPN 48.6% vs. RAPN 57.4%) achievement were comparable. On multivariate analysis, higher RENAL score was the only independent predictor of trifecta failure (OR 1.410, p=0.027). For pentafecta, higher RENAL score (OR 1.396, p=0.030), older age (OR 0.929, p=0.001), and lower pre-operative eGFR (OR 0.981, p=0.049) were independent predictors of failure.

**Conclusions:**

The study reinforced the effectiveness and safety of the new Hugo™ RAS platform for partial nephrectomy, without compromising oncological and functional success.

## INTRODUCTION

Minimally invasive partial nephrectomy (MIPN) has become the gold standard treatment for T1 RCCs and in specific cases of T2 renal masses if technically feasible, halting the development of chronic kidney disease (CKD) and the need for hemodialysis (1-4).

Robotic-assisted methods are leading the way in the advancements of kidney-sparing procedures. Robotic-assisted surgery offers superior three-dimensional (3D) visualization, tremor mitigation and better ergonomics, potentially facilitating the learning curve during the transition from laparoscopic surgery (5-7). Recent comparative studies between laparoscopic partial nephrectomy (LPN) and robot-assisted partial nephrectomy (RAPN) favor the latter due to reduced operative and warm ischemia time, shorter hospital stay, and lower conversion rates, without compromising oncological control or increasing complications (8-10).

While most studies assessing trifecta and pentafecta outcomes included patients operated by the da Vinci Surgical platform in high surgical volume centers, the generalizability of these findings to novel robotic systems remains largely unexplored. The Hugo™ RAS system was introduced as a modular robotic platform combining wristed instruments with independent robotic arm carts and an open console with 3D visualization. Although early reports suggest favorable perioperative, functional and safety outcomes, variability across platforms and the predominance of small retrospective series with unbalanced cohorts limit rigorous comparisons, underscoring the need for further studies (11-13).

We hypothesized that RAPN using the Hugo™ RAS system would provide comparable perioperative, oncological and functional results to LPN without added morbidity. Therefore, we performed a matched-pair analysis comparing these outcomes, including trifecta and pentafecta achievement and identified independent predictors of failure.

## MATERIALS AND METHODS

### Patient Selection

We retrospectively collected data on all patients eligible for minimally invasive partial nephrectomy who underwent RAPN since the introduction of the technique in the department [May 2023 to June 2025] and from a database of patients submitted to LPN [February 2015 to June 2025]. The study was approved by the institutional review board (No. 2024.061) and conducted according to the Declaration of Helsinki. A total of 165 patients were included, of which 95 and 70 underwent LPN and RAPN for renal tumors, respectively. Patients who underwent retroperitoneal PN, off-clamp or selective clamp procedures, bilateral lesions, and concomitant surgeries conducted during the same operative session were excluded from this analysis to maintain cohort homogeneity and minimize confounding variables that could influence operative time, warm ischemia time, complication rates, and length of stay.

Surgeries were performed by a team of three surgeons experienced in laparoscopic partial nephrectomy, initiating Hugo™ robot-assisted procedures. They had alternating roles between the console and the assistant surgeon. Medtronic provided in-depth technical training to the entire surgical team.

### Variables

Baseline and postoperative clinical and surgical data were collected from the patients’ health records. Evaluation of tumor complexity was based on the RENAL Nephrometry Score and stratified as low (4-6), intermediate (7-9) and high risk (10-12). The MDRD equation was applied to calculate the estimated glomerular filtration rate (eGFR) both preoperatively and at 12-months follow-up. Total operative time encompassed docking and console time. Visual estimation of blood loss (EBL) was recorded. The Clavien-Dindo classification was used to stratify postoperative complications. Trifecta outcome measurement, defined as the presence of WIT ≤ 25 min, negative surgical margins, and the absence of postoperative complications, and Pentafecta, defined as trifecta plus eGFR preservation ≥90% and absence of CKD upstaging to stage III or higher, were used to standardize the surgical success rate into an integrated scoring system.

### Surgical Technique

Patients were positioned in a 90 full flank position in LPN and 60° modified flank position for RAPN. A standardized transperitoneal approach was adopted in both groups. LPN was performed using four trocars, including a 12 mm periumbilical camera port and additional 12 mm and 5 mm working ports arranged in a triangulated configuration. In the RAPN group, an 11 mm camera port was placed along the mid-clavicular line below the costal margin. Three 8 mm robotic ports were positioned approximately 8 cm apart to prevent arm collision, with a 12 mm Air-SEAL trocar placed inferior to the camera port for the assistant. Trocar placement and docking were adjusted according to tumor laterality.

Partial nephrectomy steps were similar in both approaches (14). In summary: colon mobilization, hilar dissection, tumor identification, arterial clamping, tumor enucleation or enucleoresection, renorrhaphy with barbed suture (one or 2 layers at surgeon discretion), hemostasis verification, and specimen retrieval. A perirenal drain was routinely placed and removed when drainage was under 50cc.

### Statistical Analyses

A propensity scores matching method using the nearest-neighbor matching technique was used to address selection bias. In this regard, a multivariate logistic regression including age, gender, ASA score, BMI, preoperative eGFR, preoperative Cr, preoperative Hb, and tumor characteristics (size, laterality, RENAL nephrometry score, and clinical stage) was calculated.

Patients were matched at a 1:1 ratio, without replacement, using a caliper width of 0.20 standard deviations (SD) to minimize poor matches while preserving sample size. Patients lacking adequate covariate overlap were excluded to avoid positivity violations. Post-matching balance was assessed using standardized mean differences (threshold <0.2), variance ratios (0.5-2), and IQR ratios (0.5-2), according to variable distribution, and proportions were compared using the Chi-square test for categorical variables. Sensitivity analysis included repeated matching with alternative caliper specifications (0.15, 0.25).

Descriptive statistics were reported as mean ± SD or median and interquartile ranges (IQR) based on normality assessment and compared using independent t-test and Mann-Whitney U test, respectively. Proportions of categorical variables were assessed with the Pearson chi-square or Fisher's exact test when appropriate. Univariate and multivariate logistic regression analyses were performed to identify independent predictors of trifecta and pentafecta achievement. Data were analyzed using IBM SPSS Statistics version 31. Statistical significance was defined as p < 0.05 (two-sided).

## RESULTS

Following propensity score matching (n=140), 70 patients were included in each group. Patients’ baseline data before and after matching are summarized in Table-1. Before matching, the LPN group had a lower RENAL nephrometry score median (6 vs. 7, p=0.008), with no significant differences across remaining covariates. After matching, patients were successfully matched with an enhanced balance for all preoperative characteristics, eliminating any difference in the RENAL nephrometry score.

**Table 1:** Baseline characteristics before and after propensity score matching.

Variables		Before propensity score matching			After propensity score matching		
		**LPN (n=95)**	**RAPN (n=70)**	**P value**	**LPN (n=70)**	**RAPN (n=70)**	**P value**
Age, y, median (IQR)		63 (54-71)	64 (53-72)	0.627	63 (55-71)	65 (53-72)	0.782
Gender (male), n (%)		62 (65.3)	39 (55.7)	0.239	49 (70.0)	39 (55.7)	0.080
BMI, kg/m^2^, mean, (SD)		27.3 ± 4.5	27.2 ± 4.7	0.935	27.8 ± 4.5	27.2 ± 4.7	0.461
**ASA Score, n (%)**				**0.316**†			**0.506**†
	I	6 (6.3)	2 (2.9)		3 (4.3)	2 (2.9)	
	II	67 (69.7)	45 (64.3)		50 (71.4)	45 (64.3)	
	III	23 (24.0)	23 (32.8)		17 (24.3)	23 (32.9)	
RENAL Score, median (IQR)		6 (5-7)	7 (6-8)	**0.008**	7 (6-8)	7 (6-8)	0.130
**RENAL Score category, n (%)**				**0.083**			**0.285**
	Low (4–6)	51 (53.1)	28 (40.0)		35 (50.0)	28 (40.0)	
	Intermediate (7–9)	42 (43.8)	35 (50.0)		32 (45.7)	35 (50.0)	
	High (10–12)	3 (3.1)	7 (10.0)		3 (4.3)	7 (10.0)	
Side (Right), n (%)		45 (46.9)	28 (40.0)	0.430	36 (51.4)	27 (38.6)	0.126
Tumor Size, mm, median (IQR)		28 (20-39)	32 (21-43)	0.104	27 (21-40)	32 (22-42)	0.105
**Clinical Stage, n (%)**				**0.504**†			**0.317**†
	cT1a	74 (77.9)	54 (77.1)		53 (75.7)	54 (77.1)	
	cT1b	21 (22.1)	15 (21.5)		17 (24.3)	14 (20.0)	
	cT2a	0 (0)	1 (1.4)		0 (0)	2 (2.9)	
Pre-operative Hb, g/dL, mean (SD)		14.1 ± 1.5	14.1 ± 1.4	0.934	14.3 ± 1.5	14.1 ± 1.4	0.560
Pre-operative Cr, mg/dL, median (IQR)		0.88 (0.75–1.05)	0.84 (0.7-0.99)	0.434	0.88 (0.77-1)	0.84 (0.7-0.99)	0.498
Pre-operative eGFR, mL/min/1,73 m^2^, mean (SD)		87.2 ± 24.2	85.8 ± 21.2	0.693	87.6 ± 22.4	85.5 ± 21.6	0.561

LPN = Laparoscopic PartialNephrectomy; RAPN= Robot-Assisted PartialNephrectomy; IQR =Interquartile Range; BMI = Body Mass Index; SD = Standard Deviation; ASA = American Society of Anesthesiology; Hb = Hemoglobin; Cr = Creatinine; eGFR = Estimated Glomerular Filtration Rate.

† Fisher Exact Test

Perioperative, functional and oncological outcomes are summarized in Table-2. Operative time, WIT, and EBL were similar in both groups. Length of stay was significantly shorter in the RAPN group (3 days vs 5 days, p<0.001). Overall complication rates were higher in the LPN group, though not statistically significant (20% vs. 12.9%, p=0.178), with the majority being Clavien-Dindo I-II. When analyzing each complication, both approaches demonstrated similar incidences of urinary tract infections and postoperative fever, which were managed conservatively. Four patients (2.8%) had Clavien-Dindo III-IV complications, three in the LPN group and one in the RAPN group, all due to pseudoaneurysms requiring selective angioembolization.

**Table 2:** Perioperative, oncological and renal functional outcomes for LPN and RAPN.

Variables				
		**LPN (n=70)**	**RAPN (n=70)**	**P value**
**Perioperative data**				
Operative Time, min, median (IQR)		115 (95-141)	118 (96-145)	0.736
EBL, mL, median (IQR)		100 (50-300)	100 (50-260)	0.497
WIT, min, median (IQR)		18.5 (15.3-20)	17.3 (14.9-22)	0.594
Length of Stay, days, median (IQR)		5 (4-6)	3 (3-5)	**<0.001**
**Postoperative complications, n (%)**		**14 (20.0)**	**9 (12.9)**	**0.178**
	Clavien-Dindo I–II	11	8	
	Clavien-Dindo III–V	3	1	
Post-operative Hb, g/dL, mean (SD)		12.9 ± 1.48	12.5 ± 1.63	0.081
Post-operative Cr, mg/dL, median (IQR)		0.90 (0.8-1.2)	0.94 (0.75-1.2)	0.662
12-months follow up eGFR, mL/min/1,73 m2, mean (SD)		82.4 ± 23.4	80.3 ± 21.2	0.581
**Oncological and renal functional data**				
**Pathology, n (%)**				**0.254**†
	Benign lesions	14 (20.0)	9 (12.9)	
	Clear Cell RCC	27 (38.6)	35 (50.0)	
	Non-clear Cell RCC	29 (41.4)	26 (37.1)	
**Pathologic Stage, n (%)**				**0.542**†
	pT1a	57 (81.4)	59 (84.3)	
	pT1b	12 (17.1)	10 (14.3)	
	≥ pT2	1 (1.4)	1 (1.4)	
Positive Surgical Margin, n (%)		7 (10.0)	4 (5.7)	0.326
Trifecta Achievement, n (%)		59 (84.3)	60 (85.7)	0.813
According to RENAL Score category, n (%)*				**0.058**
	Low (4–6)	32/35 (91.4)	25/28 (89.3)	
	Intermediate (7–9)	25/32 (78.1)	30/35 (85.7)	
	High (10–12)	2/3 (66.7)	5/7 (71.4)	
	> 90% Preservation of eGFR at 12	42 (60.0)	50 (71.4)	0.154
	months follow up, n (%)			
	No CKD upstaging, n (%)	61 (87.1)	64 (91.4)	0.412
	Pentafecta Achievement, n (%)	34 (48.6)	40 (57.4)	0.310
According to RENAL Score category, n (%)*				**0.586**
	Low (4–6)	19/35 (54.3)	17/28 (60.7)	
	Intermediate (7–9)	13/32 (40.6)	19/35 (54.3)	
	High (10–12)	2/3 (66.7)	4/7 (57.1)	

LPN: Laparoscopic Partial Nephrectomy; RAPN: Robot-Assisted Partial Nephrectomy; IQR: Interquartile Range; SD: Standard Deviation; EBL: Estimated Blood Loss; WIT: Warm Ischemia Time; Hb: Hemoglobin; Cr: Creatinine; eGFR: Estimated Glomerular Filtration Rate; RCC: Renal Cell Carcinoma.

* Stratified rows show achievers/total within RENAL score category; p for trend (linear-by-linear)

‡ Fisher´s exact test

No significant differences were observed across pathological T staging distribution, histology subtypes and positive surgical margin rate. Trifecta (84.3% vs. 85.7%, p=0.813) and Pentafecta (48.6% vs. 57.4%, p=0.310) achievement were comparable, with no significant differences across RENAL score categories. During follow-up, similar rates of ≥90% eGFR preservation and absence of CKD upstaging were observed. On multivariate analysis, higher RENAL score was the only independent predictor of trifecta failure (OR 1.410, p=0.027) (Table-3). For pentafecta, older age (OR 0.929, p=0.001), higher RENAL score (OR 1.396, p=0.030), and lower pre-operative eGFR (OR 0.981, p=0.049) were independent predictors of failure (Table-4). Key perioperative outcomes are summarized in Figure-1.

**Table 3:** Univariate and multivariate logistic regression analyses for factors associated with trifecta achievement.

Variables	Univariate		Multivariate	
	**OR (95% CI)**	**P value**	**OR (95% CI)**	**P value**
Age	1.015 (0.975-1.056)	0.477	1.017 (0.978-1.058)	0.395
Gender (Female vs. Male)	0.822 (0.309-2.191)	0.695		
BMI	0.932 (0.836-1.039)	0.206		
ASA Score (III vs. I-II)	1.303 (0.483-3.514)	0.601		
Tumor size	1.025 (0.992-1.060)	0.143		
Side (right vs left)	1.132 (0.447-2.868)	0.794		
RENAL Score	1.377 (1.024-1.852)	0.034	1.410 (1.041-1.910)	**0.027**
Pre-operative eGFR	1.007 (0.985-1.029)	0.534		
EBL	1.002 (1.000-1.004)	0.125		
Operative Time	1.004 (0.991-1.018)	0.535		
Procedure (RAPN vs. LPN)	0.894 (0.353-2.263)	0.813	0.751 (0.287-1.966)	0.560

LPN = Laparoscopic Partial Nephrectomy; RAPN = Robot-Assisted Partial Nephrectomy; BMI = Body Mass Index; SD = Standard Deviation; ASA = American Society of Anesthesiology; eGFR = Estimated Glomerular Filtration Rate; OR = odds ratio; CI = Confidence interval

* Variables selected for multivariate analysis were based on statistical significance (p<0.05) and clinical relevance

**Table 4:** Univariate and multivariate logistic regression analyses for factors associated with pentafecta achievement.

Variables	Univariate		Multivariate	
	**OR (95% CI)**	**P value**	**OR (95% CI)**	**P value**
Age	0.950 (0.921-0.980)	0.001	0.929 (0.895-0.964)	**0.001**
Gender (Female vs. Male)	1.205 (0.605-2.399)	0.596		
BMI	1.017 (0.945-1.094)	0.654		
ASA Score (III vs. I-II)	1.128 (0.540-2.356)	0.748		
Tumor size	1.025 (0.992-1.060)	0.143	1.023 (0.991-1.057)	**0.167**
Side (right vs left)	0.766 (0.393-1.494)	0.794		
RENAL Score	0.870 (0.705-1.072)	0.191	1.396 (1.033-1.885)	**0.030**
Pre-operative eGFR	1.007 (0.985-1.029)	0.534	0.981 (0.962-1.000)	**0.049**
EBL	0.999 (0.997-1.001)	0.206		
Operative Time	0.986 (0.975-1.012)	0.120		
Procedure (RAPN vs. LPN)	0.894 (0.353-2.263)	0.813	1.484 (0.714-3.081)	0.290

LPN = Laparoscopic Partial Nephrectomy; RAPN = Robot-Assisted Partial Nephrectomy; BMI = Body Mass Index; SD: Standard Deviation; ASA = American Society of Anesthesiology; eGFR = Estimated Glomerular Filtration Rate; OR = odds ratio; CI = Confidence interval

* Variables selected for multivariate analysis were based on statistical significance (p<0.05) and clinical relevance.

**Figure 1:**
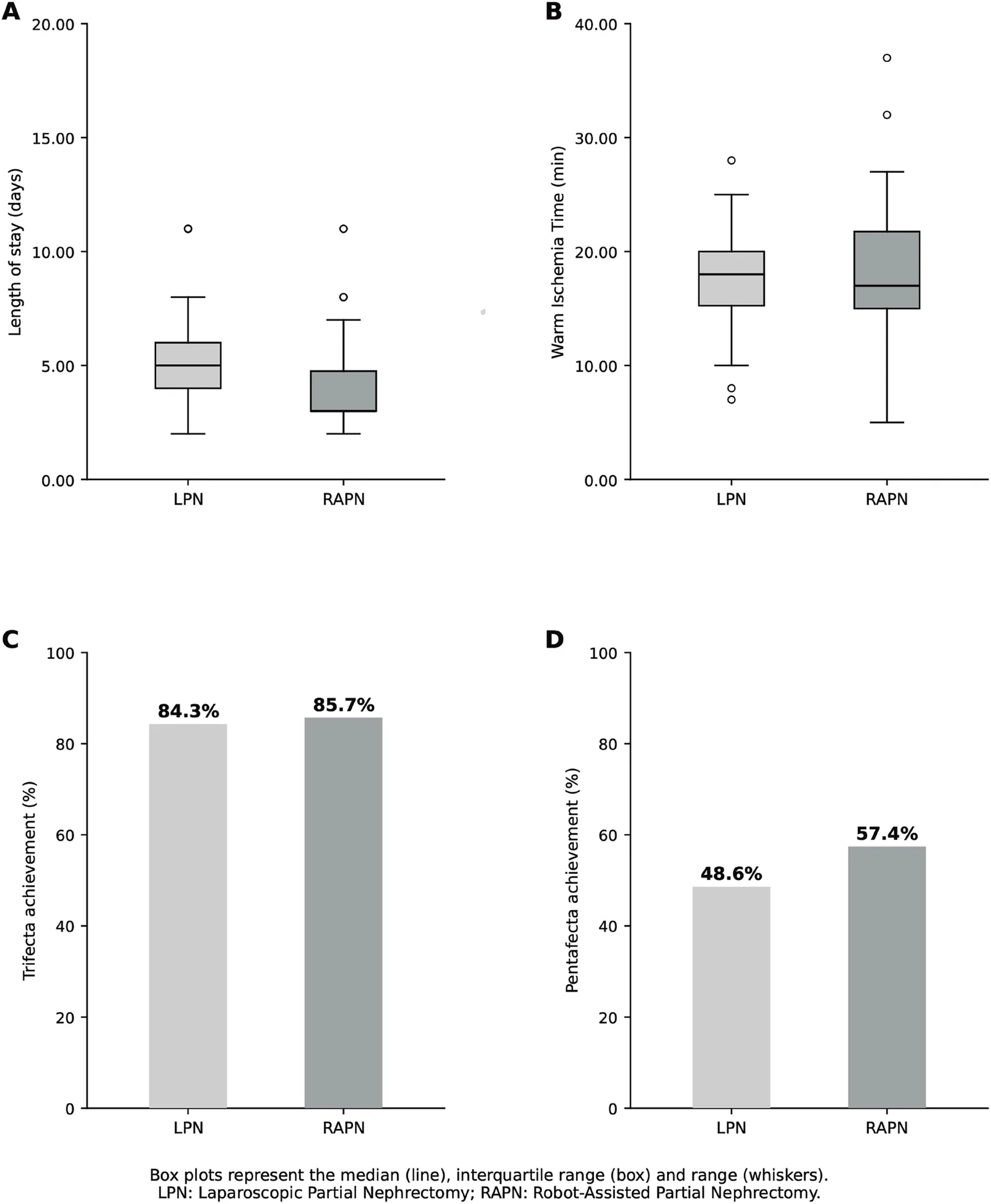
Perioperative outcomes and Trifecta/Pentafecta achievement according to surgical approach.

## DISCUSSION

Nephron-sparing surgery (NSS) has emerged as an established standard of care in the management of localized renal cell carcinoma, as preserving kidney function more effectively has the potential to halt the progression of CKD (2, 15). Additionally, NSS has been associated with a lower risk of adverse cardiovascular events by slowing atherosclerosis progression, reducing inflammatory activity, and improving endothelial function compared with radical nephrectomy. These effects lead to improved overall survival and reduced mortality (4).

Minimally invasive approaches offer lower morbidity and faster recovery while achieving comparable oncological outcomes compared to open partial nephrectomy (OPN). Several studies on RAPN have been published, demonstrating excellent perioperative and functional results, without compromising oncological success, making it the preferred approach mainly for complex renal masses (5, 13, 16-18). Few studies have assessed the performance of RAPN with the Hugo™ RAS platform.

Over the last decades, the da Vinci Surgical System™ has garnered substantial experience, leading the current evidence supporting NSS for localized RCC (19, 20). Recently, new robotic platforms have been developed, offering alternative technical features designed not only to address cost constraints but also to provide tailored solutions for challenging cases. Particularly helpful in cases of prior abdominal surgery, higher tumor complexity, or unfavorable tumor location where ergonomic systems are advantageous (21).

To date, few reports of MIPN with Hugo™ RAS system have been published; initial studies, such as Gallioli et al. and Chierigo et al. highlight some particularities of this system, such as the versatility of the modular system, 3D visualization, easier integration and direct communication within the surgical team (6, 7). They also provided valuable insight into the operative room layout and docking configurations while addressing potential setbacks.

Recent studies support smooth skill transfer and docking proficiency during the transition for surgeons with over a decade of laparoscopic experience, without compromising trifecta or pentafecta achievement. Early involvement of a consistent surgical team allows for better expertise of the technique without compromising safety (6, 22). Although modular arm carts may initially prolong docking time, workflow optimization in our series was achieved through standardized trocar placement, predefined arm cart positioning and a consistent surgical team. According to our previous experience, CUSUM analysis confirmed rapid skill acquisition with the Hugo™ RAS system, with docking proficiency achieved by the 5th case and console time stabilizing within 7–8 procedures, supporting a safe and effective transition (23). Operative time remained comparable, suggesting that docking variability did not affect surgical efficiency.

Rojo et al. conducted the first head-to-head comparison of both Da Vinci and Hugo™ RAS platforms for RAPN. As expected, docking time was shorter in the Da Vinci group (12.56min vs. 20.08min, p<0.01), reflecting the necessary adaptation and anticipated learning curve. Conversely, a significant reduction in renorrhaphy time was observed in the Hugo™ RAS group (14.33min vs. 18.94min), possibly related to improved instrument articulation and motion scaling, which may enhance suturing precision (24). Nevertheless, perioperative and oncological outcomes were comparable, without impairing safety. Although this study did not include a direct economic comparison between robotic platforms, acquisition and maintenance costs may differ. Overall financial impact remains highly dependent on institutional case volume and operating room expenses. In our institution, despite higher consumable expenses in RAPN cohort, the significant reduction in length of stay may offset this additional expenditure.

Prata et al. published the first head-to-head comparison between RAPN with Hugo™ RAS system (n=27) and LPN (n=62). They showed that the evolution from laparoscopic to robotic PN with this system did not result in increased PSM or complications; instead, it may be characterized by shorter operative times and length of stay (5). However, these findings should be interpreted with caution, owing to the heterogeneity of baseline characteristics and smaller sample size.

Recognizing these limitations, in our study, patients were matched to their nearest neighbor based on a propensity score (1:^1^), mitigating any potential baseline differences. Length of stay was significantly shorter in the RAPN group (3 days vs 5 days, p<0.001), consistent with prior reports (5, 16, 25). This difference may reflect subtle variations in early postoperative recovery not captured by the analyzed variables. Standardized postoperative pain scores were not prospectively recorded, limiting rigorous comparison, although an opioid-sparing protocol was routinely applied. Drain placement and removal protocols were standardized across both groups. Notably, both cohorts followed identical perioperative pathways, including one day prior surgery admission, ensuring institutional practices did not differentially influence the comparison. While complication rates were comparable, the higher rate observed in the LPN cohort (20% vs. 12.9%, p=0.178) may have influenced discharge timing in selected cases.

Despite comparable eGFR at 12-months follow-up, ≥90% eGFR preservation was only achieved in 60% of LPN and 71.4% of RAPN patients, highlighting that mean follow-up eGFR underestimates clinically relevant functional decline. Comparably, Wang et al. did not detect an effect of surgical approach on renal function preservation (17).

Focusing on oncological outcomes, pathological tumor stage (pT) and PSM status were comparable (10% vs. 5.7%, p=0.326), consistent with previous reports (5, 26). This highlights that RAPN is equally effective at enucleoresection with renal parenchyma preservation, without compromising adequate resection margins. Malkoc et al. showed that surgeon volume (≥25 cases/year) was a stronger predictor of surgical margin status than tumor complexity or surgical approach (27). In our series, surgeons did not reach that reported case load.

Trifecta and Pentafecta achievement represent standardized composite measures of surgical and functional success. Trifecta was comparable between groups (84.3% LPN vs. 85.7% RAPN, p=0.813) and consistent with published data, where it ranges between 60 and 90% (5, 28). However, caution is warranted when comparing series, as it lacks a standardized definition and omits some surgical factors. Furthermore, off-clamp or selective clamp procedures were not included, as surgical quality cannot be adequately assessed by trifecta. Higher RENAL score was the only independent predictor of trifecta failure in our multivariate analysis (OR 1.410, p=0.027), reflecting the association with longer WIT and greater surgical complexity (29). Tumor size alone was not an independent predictor, reflecting the predominant influence of surgical expertise over individual tumor characteristics in determining trifecta achievement.

Pentafecta was achieved in 48.6% of LPN and 57.4% of RAPN patients (p=0.310), within the range reported in the literature (29, 30). No significant differences were observed across RENAL score categories, supported by Kahn et al. (29). On multivariate analysis, Higher RENAL score (OR 1.396, p=0.030), older age (OR 0.929, p=0.001) and lower baseline eGFR (OR 0.981, p=0.049) were independent predictors of pentafecta failure, highlighting that patient-related factors appear more influential than surgical approach in determining long-term renal preservation (30).

Our study has several limitations that warrant acknowledgment. The main shortcomings are inherent to the retrospective design, conducted at a single center, with limited sample size. Longer follow-up is required to assess preserved renal parenchymal volume and long-term oncological outcomes given the recent implementation of RAPN in our institution. Furthermore, exclusion of data regarding the learning curve may have influenced postoperative outcomes. A further limitation is the different recruitment periods between cohorts, which may introduce temporal bias related to the evolving characteristics of surgical techniques and later adoption of RAPN in our institution, although propensity score matching mitigates this concern. Although ASA score was incorporated in the matching algorithm as a surrogate of comorbidity burden, validated indices such as Charlson Comorbidity Index were not available, limiting comorbidity adjustment. Furthermore, larger prospective and multicentric studies, including cost-effectiveness analyses and comparisons with other robotic platforms, are necessary to establish its long-term value.

Notwithstanding these limitations, our study is the largest head-to-head series between Hugo-RAS RAPN and LPN evaluating perioperative and functional outcomes. The propensity score matching method we employed enhances the strength of our conclusions.

## CONCLUSIONS

In conclusion, RAPN with the Hugo™ RAS system achieves comparable perioperative, trifecta and pentafecta outcomes to laparoscopy even during the early institutional adoption, with a shorter hospital stay.

## Data Availability

The original data analyzed during this study is included within this article. Data sharing is not applicable to this article

## ABBREVIATIONS

ASA: American Society of Anesthesiology
BMI: Body Mass Index
CKD: Chronic Kidney Disease
Cr: Creatinine
EBL: Estimated blood loss
eGFR: Estimated Glomerular Filtration Rate
Hb: Hemoglobin
IQR: Interquartile Ranges
LPN: Laparoscopic Partial Nephrectomy
MDRD: Modification of Diet in Renal Disease
MIPN: Minimally invasive partial nephrectomy
NSS: Nephron-sparing surgery
OT: Operative Time
PN: Partial nephrectomy
PSM: Positive surgical margins
RAPN: Robot-Assisted Partial Nephrectomy
RAS: Robot-Assisted Surgery
RCC: Renal Cell Carcinoma
SD: Standard Deviation
WIT: Warm-ischemia time
